# Medication Name Comprehension of Intelligent Virtual Assistants: A Comparison of Amazon Alexa, Google Assistant, and Apple Siri Between 2019 and 2021

**DOI:** 10.3389/fdgth.2021.669971

**Published:** 2021-05-19

**Authors:** Adam Palanica, Yan Fossat

**Affiliations:** Klick Applied Sciences, Klick Health, Klick Inc., Toronto, ON, Canada

**Keywords:** intelligent virtual assistants, voice assistants, medication names, digital medicine, verbal comprehension, speech recognition

## Abstract

The current study was a replication and comparison of our previous research which examined the comprehension accuracy of popular intelligent virtual assistants, including Amazon Alexa, Google Assistant, and Apple Siri for recognizing the generic and brand names of the top 50 most dispensed medications in the United States. Using the exact same voice recordings from 2019, audio clips of 46 participants were played back to each device in 2021. Google Assistant achieved the highest comprehension accuracy for both brand medication names (86.0%) and generic medication names (84.3%), followed by Apple Siri (brand names = 78.4%, generic names = 75.0%), and the lowest accuracy by Amazon Alexa (brand names 64.2%, generic names = 66.7%). These findings represent the same trend of results as our previous research, but reveal significant increases of ~10–24% in performance for Amazon Alexa and Apple Siri over the past 2 years. This indicates that the artificial intelligence software algorithms have improved to better recognize the speech characteristics of complex medication names, which has important implications for telemedicine and digital healthcare services.

## Introduction

Intelligent virtual (or voice) assistants (IVA), such as Amazon Alexa (hereinafter referred to as Alexa), Google Assistant, and Apple Siri (hereinafter referred to as Siri), are popular artificial intelligence (AI) software programs designed to simulate human conversation and perform web-based searches and other commands (1). Previous research has also investigated the use of these devices to gather health information and give medically related suggestions for mental and physical health inquiries (2–6). However, these findings have revealed that IVAs generally provide poor, inconsistent, and potentially harmful advice to users. A major limitation of the IVAs' ability to provide appropriate health information is their inaccurate comprehension of complex medical language syntax (7). The ability to properly recognize speech of unique words is a critical foundation of any medical interaction since any appropriate healthcare advice can only take place after the language is properly comprehended from different people.

To date, studies suggest that patients and consumers should not solely rely on IVAs to provide reliable medical information and advice. However, by definition, AI should get more “intelligent” as years pass since they are able to gather more data from users to update the software algorithms. In 2019 (7), we examined how well Alexa, Google Assistant, and Siri comprehended the generic and brand name medications of the top 50 most dispensed drugs in the United States (8, 9). Voice recordings of 46 participants (12 of which had a foreign accent in English) were played back to the IVAs. Google Assistant achieved the highest accuracy rates for brand medication names (91.8%) and generic medication names (84.3%), followed by Siri (brand names = 58.5%, generic names = 51.2%), and then by Alexa (brand names = 54.6%, generic names = 45.5%). Foreign accents also negatively affected Alexa's and Siri's medication name recognition with an ~8–11% decrease in accuracy.

Using the exact same voice recordings from 2019 (7), the current study was an identical replication to assess whether comprehension accuracy of medication names has improved over the past 2 years. Since IVAs are widely used to gather health information, it is important to examine how well the AI of these devices can upgrade for better usability in telemedicine and digital healthcare services.

## Methods

### Procedure and Data Analysis

The exact same voice recordings were used from the 46 participants who completed our previous study (7) [30 females; 16 males; age range = 23–57 years, *M* (age) = 34.2, *SD* (age) = 8.0]. The study received full ethics approval from Advarra IRB Services (www.advarra.com/services/irb-services/), and all participants signed informed written consent.

All participants spoke English fluently, with 12 participants who had various foreign accents that were different from a “Canadian accent.” Of the 12 participants with non-Canadian, foreign accents, 4 of them were United Kingdom accents, 3 Spanish/Filipino accents, 2 African accents, 2 Eastern European accents, and 1 Chinese accent.

For each generic (Supplementary Table 1) and brand (Supplementary Table 2) medication name, individual audio clips were made from participants stating the phrase “*Tell me about…*” followed by each drug name on the list (e.g., “*Tell me about acetaminophen*”).

During analysis, each voice recording was played back from a laptop using a Jabra Speak 410 speaker that was placed directly adjacent to the microphones of the IVA devices. Alexa was analyzed using a 4th-generation Amazon Echo smart speaker; Google Assistant was analyzed using a Google Pixel 4a smartphone; Siri was analyzed using an iPhone 7 smartphone. All hardware devices were updated with their latest software available, and device language was set for English (Canada). Analysis took place from December 2020 to February 2021, exactly 2 years after our previous study (7).

The primary dependent variable of comprehension was whether the IVA recognized the medication name accurately, and provided a relevant response based on the drug (i.e., accuracy %). Participants' audio clips were also scored using established norms (10, 11) as to whether the medication names were pronounced correctly or not. For statistical analyses, all of the participants' voice recordings and pronunciations were used to playback to the IVAs.

## Results

### IVA Comprehension of Medication Names

The same statistical analysis procedure was followed as our previous study (7). Comprehension accuracy rates were analyzed with a 2 (name type: brand medication, generic medication) × 3 (IVA: Alexa, Google Assistant, Siri) repeated measures analysis of variance (ANOVA), with participant accent (Canadian accent, foreign accent) as a between-subjects factor. *Post-hoc t*-tests (two-sided) were used to analyze differences in comprehension accuracy across IVAs.

A main effect of IVA was found [*F*_(2, 88)_ = 336.48, *p* < 0.0001, η_*p*_^2^ = 0.88], revealing that Google Assistant achieved the highest accuracy (*M* = 85.6%, *SD* = 9.0), which was significantly greater than Siri (*M* = 76.7%, *SD* = 11.0), which was in turn, significantly greater than Alexa (*M* = 65.4%, *SD* = 11.2).

A significant interaction between name type and IVA was also found [*F*_(2, 88)_ = 5.54, *p* = 0.008, η_*p*_^2^ = 0.11]. Paired samples *t*-tests showed that, for Siri, higher comprehension was found for brand names (*M* = 78.4%, *SD* = 8.8) than for generic names (*M* = 75.0%, *SD* = 14.5; *p* = 0.021). No significant differences were found between name types for either Alexa (brand names *M* = 64.2%, *SD* = 9.8; generic names *M* = 66.7%, *SD* = 15.1) or Google Assistant (brand names *M* = 86.0%, *SD* = 7.1; generic names *M* = 85.1%, *SD* = 12.2) (all *p* > 0.18).

No significant interactions with participant accent were found (all *p* > 0.37), which was different than our previous study that revealed more accurate responses for Siri and Alexa when comparing participants with Canadian accents to those with foreign accents (7). For comparison of total results across 2019 and 2021, Figures 1, 2 include findings from all participants (*N* = 46) and 2021, along with Canadian accent participants (*n* = 34), and foreign accent participants (*n* = 12).

**Figure 1:**
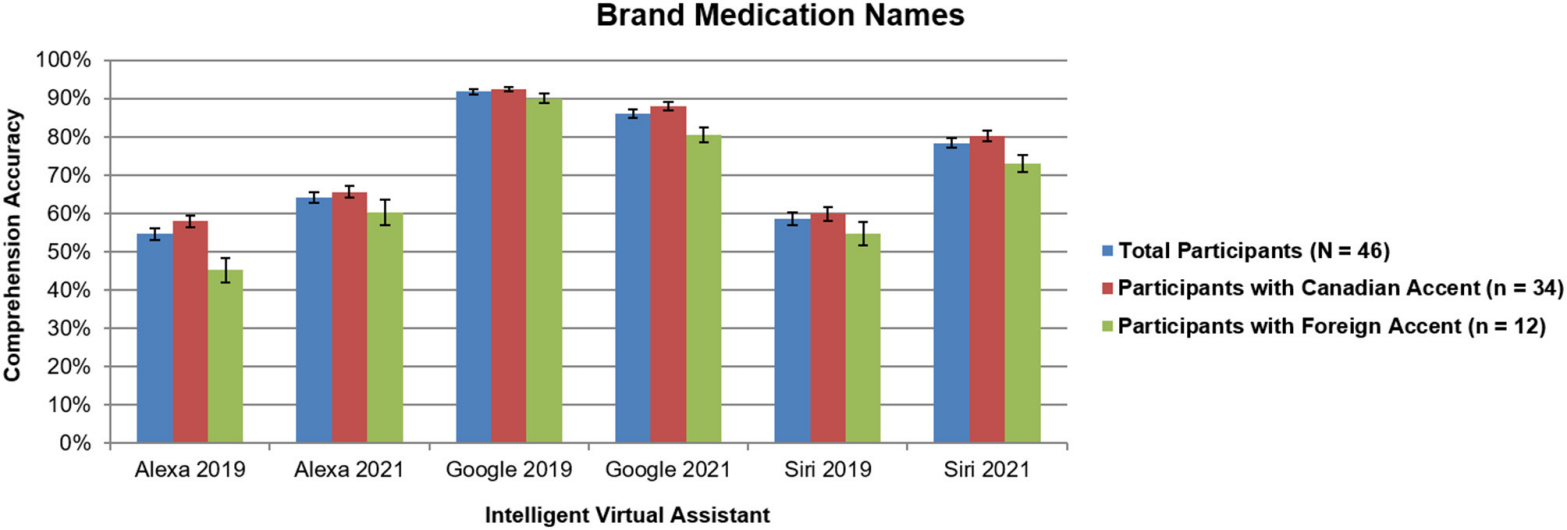
Intelligent virtual assistant comprehension accuracy of brand names for the top 50 most dispensed medications in the United States across all participants (*N* = 46) as a comparison between 2019 and 2021, shown with standard errors for each mean. Canadian accent participants (*n* = 34); foreign accent participants (*n* = 12). Percentages represent the average accuracy rates for participants across all 50 medication names. See text for more details.

**Figure 2:**
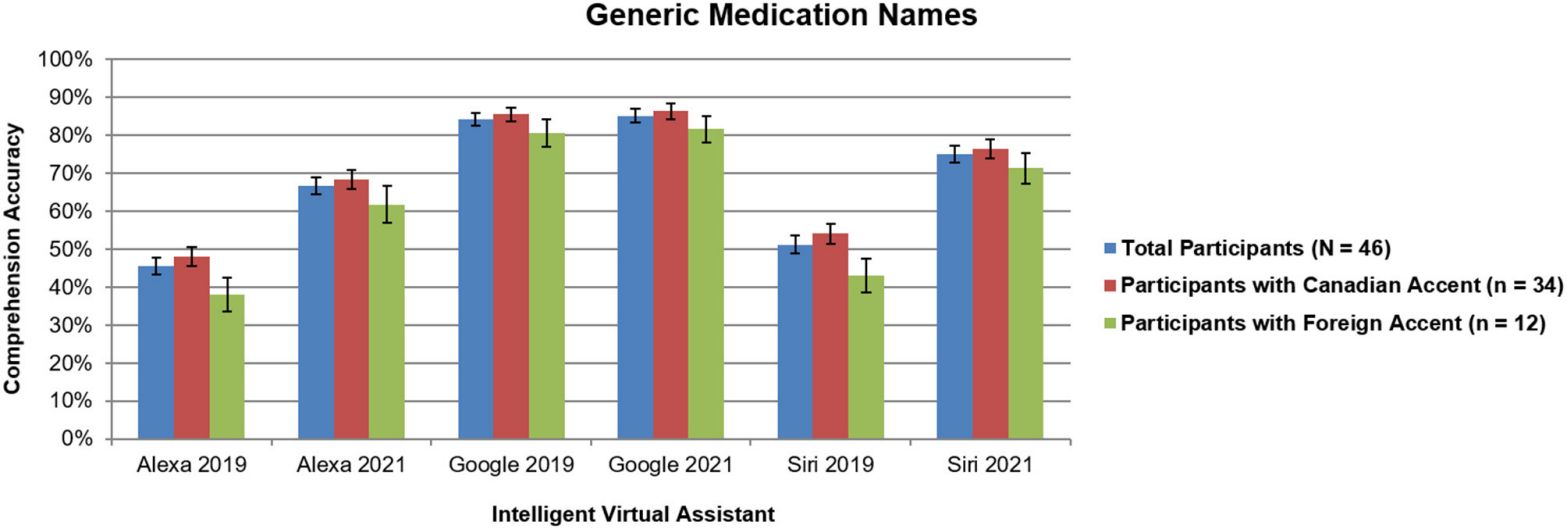
Intelligent virtual assistant comprehension accuracy of generic names for the top 50 most dispensed medications in the United States across all participants (*N* = 46) as a comparison between 2019 and 2021, shown with standard errors for each mean. Canadian accent participants (*n* = 34); foreign accent participants (*n* = 12). Percentages represent the average accuracy rates for participants across all 50 medication names. See text for more details.

Similar to 2019 (7), analyses revealed no significant effects of participant age or gender on comprehension accuracy, and no significant correlations were found between dispense rates (i.e., a proxy of commonality) (9) or the amount of syllables in each word for either brand or generic medication comprehension accuracy across any of the IVAs.

### IVA Comprehension Comparison Between 2019 and 2021

Paired samples *t*-tests (two-sided) were used to analyze differences in comprehension accuracy across IVAs between 2019 and 2021, for total participants and across different participant accent groups.

For brand medication names (Figure 1), comprehension for Alexa and Siri significantly increased, whereas for Google Assistant, accuracy significantly decreased (all *p* < 0.0001). This was the case for both Canadian accent participants and foreign accent participants.

For generic medication names (Figure 2), comprehension for Alexa and Siri significantly increased (all *p* < 0.0001), whereas for Google Assistant, no significant difference was found (*p* > 0.19). This effect was consistent for both Canadian accent participants and foreign accent participants.

## Discussion

This study was an identical replication of our previous research (7) assessing how well Alexa, Google Assistant, and Siri comprehended brand name and generic name medications of the 50 most dispensed drugs in the United States. Using the same voice recordings from 2019, we played back each medication name audio clip into the latest IVA technology in 2021 to evaluate any improvements in comprehension accuracy.

Similar to 2019, this study demonstrated that Google Assistant yielded the highest comprehension accuracy for both brand medication names (86.0%) and generic medication names (84.3%); Siri came in second place with significantly lower comprehension performance for brand names (78.4%) and generic names (75.0%), followed by Alexa in last place, which yielded the worst performance for brand names (64.2%) and generic names (66.7%).

Although the overall trends were the same as our previous study (7), Figures 1, 2 reveal dramatic increases of ~10–24% in performance for Alexa and Siri over the past 2 years, closely approaching the performance of Google Assistant. This shows that the AI software algorithms have improved over the past 2 years to better recognize the speech characteristics of complex medication names. Additionally, our previous study demonstrated a main effect of name type, with significant increases in accuracy for brand names vs. generic names across all three IVAs (7); however, in the current study, only a slight difference in performance was found between brand and generic drug names for Siri, with no differences for Alexa or Google Assistant. This finding indicates that the gap is closing in terms of comprehending syntax of medication language.

The current study also found no significant interactions of participant accent in comprehension accuracy. By contrast, our previous study revealed an ~8–11% difference in accuracy for Siri and Alexa when comparing participants with Canadian accents to those with foreign accents (7). This indicates an overall improvement of AI technology becoming more usable in everyday situations across a variety of user demographics.

Google Assistant remains the best of the three IVAs for comprehending brand and generic drug names. No differences were found for generic names between 2019 and 2021, possibly indicating a ceiling effect in performance. Counterintuitively, for brand names, comprehension accuracy decreased by almost 6% over the past 2 years. Perhaps this is because brand names (compared to generic names) are more likely to have homophones associated with them in the English language (e.g., Singulair vs. “singular,” or Bayer vs. “bear”). Consequently, Google Assistant may comprehend words based on the subtlest nuances of annunciations, so if a participant mispronounces a brand name, Google misrecognizes the word to be more like it sounds rather than a specific medication.

Similar to our previous paper (7), some limitations should be noted. The current study did not assess the usefulness or safety of the medical information given, as some other previous research has examined (2–6), although this was not the primary purpose of the study. Future research could investigate the implications of errors when comprehending medication names during interactions with different patient conditions, symptoms, contraindications, and side effects. Additionally, this study did not include an exhaustive set of all possible ethic accents, and only focused on commonly dispensed medications in the United States. Future research could examine other real-world implications of IVA comprehension in different countries and languages when gathering medical information.

Overall, this study demonstrated that IVA technology and AI software is indeed improving for recognizing medication names, and may even be reaching a ceiling effect in performance relative to Google Assistant. Comprehension accuracy of IVAs within healthcare has extremely important implications with respect to delivering appropriate medical information to patients, especially to individuals with reduced ability to read small font of medication labels or type on a mobile device (12). Additionally, given the importance of remote healthcare delivery and telemedicine services during a global pandemic, IVAs are becoming more important to provide critical healthcare to patients everywhere (13). With the rise of IVA home use and advancement of AI in general, this research provides a vital foundation for systemic comparison of the longitudinal improvement of technology, with implications across patients of all demographics.

## Data Availability Statement

The raw data supporting the conclusions of this article will be made available by the authors, without undue reservation.

## Ethics Statement

The studies involving human participants were reviewed and approved by Advarra IRB Services (http://www.advarra.com/services/irb-services/). The patients/participants provided their written informed consent to participate in this study.

## Author Contributions

AP analyzed the data and contributed to writing the manuscript with assistance by YF. All authors contributed to the final review and editing, and have approved the final manuscript.

## Conflict of Interest

AP and YF were employed by the company Klick Health. The authors declare that the research was conducted in the absence of any commercial or financial relationships that could be construed as a potential conflict of interest.
