# Supplementary material for: Medication Name Comprehension of Intelligent Virtual Assistants: A Comparison of Amazon Alexa, Google Assistant, and Apple Siri Between 2019 and 2021
Source: Front Digit Health. 2021 May 19;3:669971. doi: 10.3389/fdgth.2021.669971 (PMC8521933; doi:10.3389/fdgth.2021.669971)
Supplement: Supplementary file 1 [file Table_1.DOCX]

| Supplementary Table 1. Generic Names of the Top 50 Most Dispensed Medications in the United States, in Alphabetical Order, with Relative Comprehension Accuracy for Each Name (N = 46) | | | | | | |
| --- | --- | --- | --- | --- | --- | --- |
|  | Alexa Comprehension Accuracy Mean % | | Google Assistant Comprehension Accuracy Mean % | | Siri Comprehension Accuracy Mean % | |
| Medications | 2019 | 2021 | 2019 | 2021 | 2019 | 2021 |
| 1. Acetaminophen | 59% | 80% | 80% | 85% | 72% | 85% |
| 1. Albuterol | 41% | 72% | 83% | 83% | 67% | 72% |
| 1. Alprazolam | 52% | 83% | 89% | 87% | 74% | 83% |
| 1. Amlodipine | 41% | 70% | 80% | 78% | 50% | 57% |
| 1. Amoxicillin | 80% | 96% | 98% | 100% | 83% | 100% |
| 1. Aspirin | 93% | 100% | 100% | 100% | 93% | 100% |
| 1. Atenolol | 37% | 54% | 80% | 83% | 57% | 76% |
| 1. Atorvastatin | 33% | 52% | 65% | 78% | 43% | 70% |
| 1. Azithromycin | 46% | 74% | 70% | 78% | 28% | 59% |
| 1. Bupropion | 24% | 35% | 61% | 61% | 33% | 57% |
| 1. Carvedilol | 43% | 65% | 76% | 83% | 41% | 54% |
| 1. Citalopram | 0% | 39% | 85% | 91% | 33% | 65% |
| 1. Clonazepam | 57% | 80% | 89% | 91% | 61% | 83% |
| 1. Clopidogrel | 15% | 61% | 59% | 76% | 24% | 50% |
| 1. Cyclobenzaprine | 74% | 93% | 96% | 98% | 83% | 93% |
| 1. Duloxetine | 15% | 57% | 78% | 80% | 39% | 59% |
| 1. Escitalopram | 24% | 24% | 50% | 54% | 4% | 43% |
| 1. Ethinylestradiol | 26% | 33% | 48% | 59% | 26% | 41% |
| 1. Fluoxetine | 33% | 43% | 89% | 76% | 59% | 78% |
| 1. Fluticasone | 22% | 46% | 65% | 74% | 37% | 48% |
| 1. Furosemide | 41% | 59% | 83% | 85% | 43% | 63% |
| 1. Gabapentin | 85% | 91% | 96% | 98% | 89% | 93% |
| 1. Glipizide | 43% | 74% | 78% | 74% | 50% | 74% |
| 1. Hydrochlorothiazide | 63% | 85% | 100% | 100% | 65% | 80% |
| 1. Hydrocodone | 96% | 98% | 100% | 100% | 85% | 98% |
| 1. Ibuprofen | 4% | 93% | 98% | 100% | 89% | 100% |
| 1. Insulin Glargine | 11% | 2% | 89% | 91% | 39% | 61% |
| 1. Levothyroxine | 7% | 54% | 80% | 80% | 15% | 26% |
| 1. Lisinopril | 11% | 37% | 78% | 85% | 39% | 91% |
| 1. Losartan | 46% | 80% | 83% | 83% | 20% | 91% |
| 1. Meloxicam | 70% | 83% | 89% | 96% | 39% | 91% |
| 1. Metformin | 59% | 83% | 87% | 87% | 43% | 72% |
| 1. Metoprolol | 17% | 26% | 87% | 83% | 7% | 65% |
| 1. Montelukast | 70% | 89% | 93% | 93% | 13% | 87% |
| 1. Omeprazole | 50% | 67% | 96% | 76% | 43% | 78% |
| 1. Oxycodone | 96% | 96% | 98% | 98% | 96% | 96% |
| 1. Pantoprazole | 24% | 89% | 93% | 96% | 67% | 89% |
| 1. Potassium Chloride | 100% | 100% | 100% | 100% | 74% | 98% |
| 1. Pravastatin | 46% | 46% | 96% | 89% | 28% | 80% |
| 1. Prednisone | 70% | 85% | 91% | 93% | 74% | 85% |
| 1. Ranitidine | 17% | 37% | 83% | 76% | 37% | 72% |
| 1. Rosuvastatin | 0% | 48% | 48% | 70% | 24% | 52% |
| 1. Sertraline | 39% | 61% | 87% | 76% | 50% | 74% |
| 1. Simvastatin | 48% | 70% | 100% | 100% | 30% | 76% |
| 1. Tamsulosin | 50% | 46% | 78% | 76% | 59% | 83% |
| 1. Tramadol | 89% | 96% | 100% | 100% | 91% | 98% |
| 1. Trazodone | 61% | 87% | 98% | 96% | 83% | 85% |
| 1. Venlafaxine | 54% | 72% | 76% | 72% | 52% | 72% |
| 1. Warfarin | 41% | 46% | 98% | 93% | 59% | 76% |
| 1. Zolpidem | 54% | 78% | 89% | 76% | 50% | 74% |

| Supplementary Table 2. Brand Names of the Top 50 Most Dispensed Medications in the United States, Ordered in Reference to Supplementary Table 1, with Relative Comprehension Accuracy for Each Name (N = 46) | | | | | | |
| --- | --- | --- | --- | --- | --- | --- |
|  | Alexa Comprehension Accuracy Mean % | | Google Assistant Comprehension Accuracy Mean % | | Siri Comprehension Accuracy Mean % | |
| Medications | 2019 | 2021 | 2019 | 2021 | 2019 | 2021 |
| 1. Tylenol | 98% | 91% | 98% | 100% | 93% | 100% |
| 1. Ventolin | 70% | 0% | 100% | 100% | 67% | 87% |
| 1. Xanax | 87% | 89% | 98% | 96% | 93% | 93% |
| 1. Norvasc | 70% | 87% | 91% | 89% | 72% | 83% |
| 1. Amoxil | 35% | 52% | 91% | 89% | 13% | 59% |
| 1. Bayer | 7% | 0% | 83% | 9% | 17% | 0% |
| 1. Tenormin | 30% | 37% | 80% | 70% | 20% | 61% |
| 1. Lipitor | 89% | 87% | 100% | 98% | 87% | 93% |
| 1. Zithromax | 52% | 74% | 98% | 91% | 67% | 72% |
| 1. Wellbutrin | 80% | 59% | 96% | 91% | 7% | 61% |
| 1. Coreg | 59% | 4% | 96% | 65% | 9% | 4% |
| 1. Celexa | 87% | 96% | 100% | 100% | 89% | 98% |
| 1. Klonopin | 74% | 65% | 100% | 93% | 72% | 93% |
| 1. Plavix | 37% | 57% | 100% | 91% | 67% | 89% |
| 1. Flexeril | 70% | 87% | 96% | 98% | 89% | 91% |
| 1. Cymbalta | 83% | 76% | 96% | 93% | 83% | 93% |
| 1. Lexapro | 59% | 98% | 100% | 100% | 93% | 98% |
| 1. Ortho Tri-Cyclen | 0% | 91% | 98% | 93% | 93% | 93% |
| 1. Prozac | 98% | 96% | 100% | 100% | 96% | 98% |
| 1. Flonase | 78% | 83% | 96% | 89% | 83% | 78% |
| 1. Lasix | 63% | 61% | 80% | 67% | 43% | 80% |
| 1. Neurontin | 57% | 57% | 93% | 91% | 63% | 78% |
| 1. Glucotrol | 43% | 0% | 80% | 83% | 43% | 83% |
| 1. Microzide | 33% | 80% | 48% | 76% | 54% | 93% |
| 1. Vicodin | 74% | 89% | 96% | 89% | 33% | 89% |
| 1. Advil | 2% | 93% | 100% | 98% | 59% | 98% |
| 1. Lantus | 39% | 96% | 100% | 91% | 11% | 54% |
| 1. Levaquin | 76% | 74% | 100% | 98% | 80% | 93% |
| 1. Prinivil | 11% | 67% | 93% | 83% | 33% | 83% |
| 1. Cozaar | 2% | 85% | 96% | 87% | 4% | 78% |
| 1. Mobic | 85% | 93% | 100% | 100% | 67% | 98% |
| 1. Glucophage | 74% | 89% | 98% | 96% | 85% | 91% |
| 1. Lopressor | 78% | 96% | 100% | 93% | 57% | 91% |
| 1. Singulair | 67% | 76% | 100% | 98% | 52% | 65% |
| 1. Prilosec | 59% | 78% | 87% | 63% | 46% | 78% |
| 1. Oxycontin | 98% | 100% | 100% | 100% | 72% | 72% |
| 1. Protonix | 78% | 85% | 96% | 96% | 83% | 89% |
| 1. K-Dur | 0% | 0% | 0% | 0% | 0% | 0% |
| 1. Pravachol | 20% | 0% | 89% | 78% | 24% | 63% |
| 1. Deltasone | 11% | 0% | 91% | 80% | 33% | 52% |
| 1. Zantac | 89% | 93% | 100% | 96% | 87% | 98% |
| 1. Crestor | 70% | 52% | 100% | 93% | 65% | 98% |
| 1. Zoloft | 80% | 85% | 100% | 96% | 91% | 93% |
| 1. Zocor | 78% | 80% | 96% | 89% | 70% | 74% |
| 1. Flomax | 93% | 85% | 100% | 100% | 98% | 100% |
| 1. Ultram | 4% | 43% | 100% | 98% | 20% | 72% |
| 1. Desyrel | 11% | 20% | 41% | 33% | 22% | 30% |
| 1. Effexor | 7% | 24% | 91% | 83% | 63% | 93% |
| 1. Coumadin | 43% | 50% | 98% | 89% | 59% | 85% |
| 1. Ambien | 24% | 28% | 100% | 100% | 100% | 100% |
